# Effects of Microwave-Assisted Extraction Conditions on Antioxidant Capacity of Sweet Tea (*Lithocarpus polystachyus* Rehd.)

**DOI:** 10.3390/antiox9080678

**Published:** 2020-07-29

**Authors:** Ao Shang, Min Luo, Ren-You Gan, Xiao-Yu Xu, Yu Xia, Huan Guo, Yi Liu, Hua-Bin Li

**Affiliations:** 1 Guangdong Provincial Key Laboratory of Food, Nutrition and Health, Department of Nutrition, School of Public Health, Sun Yat-Sen University, Guangzhou 510080, China; shangao@mail2.sysu.edu.cn (A.S.); luom65@mail2.sysu.edu.cn (M.L.); xuxy53@mail2.sysu.edu.cn (X.-Y.X.); 2 National Agricultural Science & Technology Center, Chengdu 610213, China; ganrenyou@caas.cn (R.-Y.G.); xiayu01@caas.cn (Y.X.); ghscny@163.com (H.G.); liuyi03@caas.cn (Y.L.); 3 Research Center for Plants and Human Health, Institute of Urban Agriculture, Chinese Academy of Agricultural Sciences, Chengdu 610213, China

**Keywords:** sweet tea, *Lithocarpus polystachyus* Rehd., antioxidant, microwave-assisted extraction, response surface methodology

## Abstract

In this study, the effects of microwave-assisted extraction conditions on antioxidant capacity of sweet tea (*Lithocarpus polystachyus* Rehd.) were studied and the antioxidants in the extract were identified. The influences of ethanol concentration, solvent-to-sample ratio, microwave power, extraction temperature and extraction time on Trolox equivalent antioxidant capacity (TEAC) value, ferric reducing antioxidant power (FRAP) value and total phenolic content (TPC) were investigated by single-factor experiments. The response surface methodology (RSM) was used to study the interaction of three parameters which had significant influences on antioxidant capacity including ethanol concentration, solvent-to-sample ratio and extraction time. The optimal conditions for the extraction of antioxidants from sweet tea were found as follows—ethanol concentration of 58.43% (*v*/*v*), solvent-to-sample ratio of 35.39:1 mL/g, extraction time of 25.26 min, extraction temperature of 50 ℃ and microwave power of 600 W. The FRAP, TEAC and TPC values of the extract under the optimal conditions were 381.29 ± 4.42 μM Fe(II)/g dry weight (DW), 613.11 ± 9.32 μM Trolox/g DW and 135.94 ± 0.52 mg gallic acid equivalent (GAE)/g DW, respectively. In addition, the major antioxidant components in the extract were detected by high-performance liquid chromatography with diode array detection (HPLC-DAD), including phlorizin, phloretin and trilobatin. The crude extract could be used as food additives or developed into functional food for the prevention and management of oxidative stress-related diseases.

## 1. Introduction

Sweet tea comes from the leaves of the evergreen tree *Lithocarpus polystachyus* Rehd. and its taste is sweet [1]. Sweet tea has already been approved as new food materials in China since 2017. Sweet tea contains various bioactive ingredients, such as polyphenols and polysaccharides, especially flavonoids [2]. The main flavonoids in sweet tea are dihydrochalcones, including phlorizin, phloretin and trilobatin. These compounds are the main bioactive components of sweet tea and also major contributors to its sweetness [3]. Sweet tea and its bioactive components have been found to have antioxidant, antimicrobial, anti-inflammatory, antidiabetic, anticancer and cardiovascular protective activities [4,5,6,7,8,9]. The effective extraction of bioactive components in sweet tea is helpful for its exploitation and utilization.

In general, traditional extraction methods, such as maceration, percolation and Soxhlet extraction, are most commonly used methods to extract phytochemicals [10,11]. However, these traditional methods have the disadvantages of the large consumption of organic solvent, long extract time and low efficiency [12,13]. Therefore, some new extraction methods came into being to overcome these problems, including ultrasound-assisted extraction, pressurized liquid extraction, enzyme-assisted extraction and microwave-assisted extraction (MAE) [14,15,16]. Microwave-assisted extraction has been applied in the extraction of some plant active compounds. Microwave radiation has a destructive effect on cell structure, which makes the active substance dissolve into the solvent quickly, thus obtaining higher extraction efficiency in a shorter time [17]. At the same time, the microwave-assisted extraction also has strong advantages in stability and reproducibility [18]. To our knowledge, no report could be found in the literature on the extraction of antioxidants from sweet tea by microwave-assisted extraction.

The efficiency of microwave-assisted extraction is affected by many factors, including microwave power, extraction time, temperature, solvent-to-sample ratio and solvent concentration and there are also interactions between these parameters [19,20,21]. The response surface methodology (RSM) can be applied to analyze the relationship between response values and multiple variables by establishing a fitting model [22]. Therefore, the RSM was conducted to study the influence of various factors on the efficiency of extracting antioxidants from sweet tea and the interaction among them, so as to obtain the optimal extraction conditions.

In this study, we evaluated the antioxidant activity of sweet tea extracts by ferric-reducing antioxidant power (FRAP) and Trolox equivalent antioxidant capacity (TEAC) assays and measured the total phenol content (TPC) of the extracts. The single-factor test was performed to investigate the effects of microwave power, extraction time, extraction temperature, solvent concentration and solvent-to-sample ratio on the extraction efficiency. Then three variables with obvious effects were selected for the RSM and the optimal extraction conditions were subsequently verified. Also, the extraction efficiency of microwave-assisted extraction was compared with that of the maceration method. In addition, the main compounds, phlorizin, phloretin and trilobatin in the extract were analyzed qualitatively and quantitatively by high-performance liquid chromatography with diode array detection (HPLC-DAD).

## 2. Materials and Methods

### 2.1. Plant Sample and Reagents

*L. polystachyus* Rehd. leaves were collected from Ya’an, Sichuan Province, China. The *L. polystachyus* leaves were washed and then dried at 60 °C for 12 h and the moisture content was 0.56 ± 0.45%. The dried leaves were ground into powder with a grinder (RS-FS500B; Royalstar Co., Ltd., Hefei, China), then filtered through a 100-mesh sieve. The powder of leaves were sealed and stored at 4 °C.

The chemicals 6-hydroxy-2,5,7,8-tetramethylchromane-2-carboxylic acid (Trolox), 2,2′-azinobis (3-ethylbenothiazoline-6-sulfonic acid) diammonium salt (ABTS), 2,4,6-tri(2-pyridyl)-*S*-triazine (TPTZ), Folin–Ciocalteu’s phenol reagent, gallic acid were purchased from Sigma-Aldrich (St. Louis, MO, USA). The standards phloridzin and phloretin for HPLC-DAD analysis were obtained from Ark Pharm, Inc. (Libertyville, IL, USA). The standard trilobatin was purchased from Shanghai Yi En chemical technology Co., Ltd. (Shanghai, China). The formic acid and methanol were of chromatographic grade and obtained from Macklin Chemical Factory (Shanghai, China). The ethanol, sodium acetate, acetic acid, hydrochloric acid, iron(III) chloride hexahydrate, iron(II) sulfate heptahydrate, potassium persulfate and sodium carbonate were of analytical grade and purchased from Tianjin Chemical Factory (Tianjin, China). Deionized water was used for all experiments.

### 2.2. Microwave-Assisted Extraction (MAE)

The MAE process was performed by microwave equipment with controlled temperature, extraction time and microwave power (X-100A; Xianghu Instrumental Company, Beijing, China). Dried *L. polystachyus* leaf sample (1.000 g) was placed in a centrifuge tube and then mixed with ethanol aqueous solutions of different concentrations and volumes. After extraction, the mixture was cooled with running water, centrifuged at 4200× *g* for 8 min and the supernatant was collected for subsequent experiments.

### 2.3. Maceration Extraction (ME)

The leaf sample (1.000 g) was immersed in 35.39 mL of 58.43% (*v*/*v*) ethanol aqueous solution and extracted in a water bath shaker at 25 ℃ for 24 h. After centrifugation of the mixture (4200× *g*, 8 min), the supernatant was collected.

### 2.4. Antioxidant Activity Assays

In this study, the ferric reducing antioxidant power (FRAP) and Trolox equivalent antioxidant capacity (TEAC) assays were adopted to determine the antioxidant activity of the extract of *L. polystachyus* leaf and the procedure was based on the literature [16]. TEAC assay was used to measure the free radical scavenging ability of the extract and FRAP assay was used to test its Fe(III) reducing capacity [23].

#### 2.4.1. Trolox Equivalent Antioxidant Capacity (TEAC) Assay

ABTS^+^ stock solution is composed of an equal volume of 7 mmol/L ABTS^+^ solution and 2.45 mmol/L potassium persulfate solution and it is used after 16 h of light-avoiding incubation, effective within 48 h. The absorbance of ABTS^+^ stock solution was diluted to 0.71 ± 0.05 at 734 nm and the dilution multiple was calculated to prepare the ABTS^+^ reaction solution. The 100 μL diluted supernatant was thoroughly mixed with 3.8 mL ABTS^+^ reaction solution and the reaction was conducted at room temperature for 6 min in the dark. Then the absorbance of the mixture at 734 nm was measured and the absorbance inhibition percentage was calculated. The standard curve was carried out with different concentrations of standard Trolox solution and the final TEAC value was represented by μmol Trolox/g dry weight (DW) of *L. polystachyus* leaf.

#### 2.4.2. Ferric Reducing Antioxidant Power (FRAP) Assay

The 300 mmol/L of sodium acetate buffer, 10 mmol/L of TPTZ solution and 20 mmol/L of ferric chloride solution were mixed at a volume ratio of 10:1:1 to obtain the FRAP working solution. The 100 μL diluted supernatant (diluted with the ethanol solution of the same concentration as the extraction solvent) was added to 3 mL FRAP reaction solution for 4 min and its absorbance at 593 nm was tested. The ferrous sulfate was applied to make a standard curve and the FRAP value was shown as µmol Fe(II)/g DW of *L. polystachyus* leaf.

### 2.5. Determination of Total Phenolic Content (TPC)

The Total Phenolic Content (TPC) was detected by the Folin–Ciocalteu method according to previous literature [24]. The 500 μL diluted supernatant was added to 2.5 mL 0.2 mol/L Folin-Ciocalteu reagent, reacted for 4 min and then added 2 mL saturated sodium carbonate solution. The mixture was incubated at room temperature in the dark for 2 h and the absorbance was detected at 760 nm. The standard curve of gallic acid was drawn and mg gallic acid equivalent (GAE)/g DW of *L. polystachyus* leaf was used to express TPC.

### 2.6. Experimental Design and Statistical Analysis

#### 2.6.1. Single-Factor Tests

Effects of five independent process parameters on extraction efficiency were tested, including ethanol concentration (20–80%, *v*/*v*), solvent-to-sample ratio (10:1–60:1 mL/g), extraction time (10–60 min), extraction temperature (30–70 °C) and microwave power (400–900 W). With other parameters fixed, the change of one factor was observed to investigate its influence on antioxidant activity and TPC of the samples and three main influencing factors were obtained.

#### 2.6.2. Response Surface Methodology (RSM)

In this study, the RSM based on a three-variable, five-level central composite design (CCD) was used to optimize the extraction condition of *L. polystachyus* leaf. The three major variables obtained from single-factor tests, including solvent-to-sample ratio, extraction time and ethanol concentration, were regarded as independent variables of CCD and each variable was encoded into 5 levels of −1.682, −1, 0, 1 and 1.682, respectively (Table 1).

The CCD matrix contains 20 experiments with 6 replicates of center points. The response values of the model were analyzed by Design-Expert 8.0.6 and the data were subjected to multiple regression analysis to fit the quadratic equation:

(1) $$Y=\beta_{0}+\overset{n}{\underset{i=1}{\sum }}\beta_{i}X_{i}\;+\overset{n-1}{\underset{i=1}{\sum }}\overset{n}{\underset{j=2}{\sum }}\beta_{\mathrm{ij}}X_{i}X_{j}\;+\overset{n}{\underset{i=1}{\sum }}\beta_{\mathrm{ii}}X_{i}^{2}+\varepsilon ,$$

where Y refers to the predicted response values of TEAC, FRAP and TPC; β_0_ is the constant coefficient; β_i_, β_ij_ and β_ii_ represent the coefficients of linear, quadratic and interaction terms, respectively; X_i_ and X_j_ are independent variables; ε is the residual error.

Analysis of variance (ANOVA) was used to analyze the statistical significance of the fitting model and each term of the fitted model. The interaction effect of each variable on the response value was shown on the 3D surface plot. Finally, the theoretical optimal extraction conditions and response values were verified.

### 2.7. High-Performance Liquid Chromatography with Diode Array Detection (HPLC-DAD) Analysis

The main components in the extract of *L. polystachyus*, including phloridzin, phloretin and trilobatin, were determined by the HPLC-DAD method. The HPLC analysis was conducted on a Waters HPLC system (Milford, MA, USA), which was composed of a Waters 1525 binary HPLC pump with an auto-injector, a Waters 2996 photodiode array detector and an Agilent Zorbax Eclipse XDB-C18 column (250 mm × 4.6 mm, 5 µm) (Santa Clara, CA, USA). The mobile phase consisted of methanol (solution A) and 0.1% formic acid (solution B), at a flow rate of 0.8 mL/min and the elution procedure is shown in Table 2. The column temperature was set at 35 °C and the injection volume was 20 μL. According to the previous literatures, the wavelength of diode array detector was set at 283 nm [25,26]. The target compounds in sweet tea were qualitatively analyzed by comparing with the retention time and spectra of the standards and quantitatively analyzed by comparing with the peak area at the maximum absorption wavelength of the standards with different concentrations. The results were expressed as mg/g DW of sweat tea.

### 2.8. Statistical Analysis

All experiments were performed in triplicate and the results were displayed as mean ± standard deviation. The data were analyzed by Design Expert 10 software (Stat-Ease Inc., Minneapolis, MN, USA) and SPSS 26.0 statistics software (IBM Corp., Armonk, NY, USA). The statistical significance was investigated by one-way ANOVA and the significance level was *p* < 0.05.

## 3. Results and Discussion

### 3.1. Analysis of Single-Factor Tests

#### 3.1.1. Effects of Ethanol Concentration

**Figure 1:**
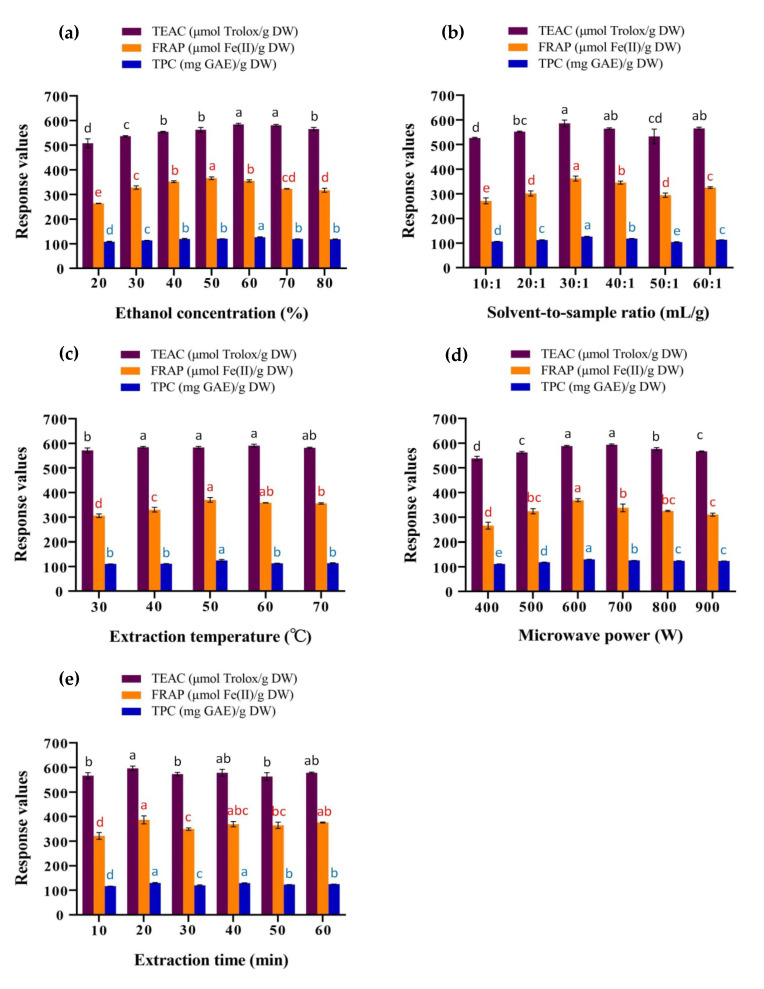
Effects of single factors on the Trolox equivalent antioxidant capacity (TEAC), ferric reducing antioxidant power (FRAP) and total phenolic content (TPC) values of *L. polystachyus* leaf extract: ethanol concentration (**a**), solvent-to-sample ratio (**b**), extraction temperature (**c**), microwave power (**d**) and extraction time (**e**). One-way ANOVA was used to compare the significant differences among groups. Different letters (a, b, c, d, e) in black, red and blue colors respectively represent significant differences (*p* < 0.05) in TEAC, FRAP and TPC values among groups. The same letter indicates no significant difference (*p* > 0.05) among groups.

Ethanol is a common polar solvent and is relatively safe for human beings. The ethanol aqueous solution is widely used in the extraction of antioxidants from plants and the ethanol concentration has a certain effect on the extraction efficiency [27,28]. Other conditions remained the same (500 W, 50 °C, 30:1 mL/g and 30 min), the trend of the antioxidant activity and TPC of the extracts as a function of ethanol concentration is shown in Figure 1a.

As shown in Figure 1a, the TEAC values of the extracts kept increasing between 20–60% (*v*/*v*) of the ethanol concentration and then decreased slower. At the same time, FRAP values were observed to rise between 20–50% (*v*/*v*) of ethanol concentration and fall from 50% (*v*/*v*). The TPCs of the extracts showed the same trend as TEAC values, with the highest point was at 60% (*v*/*v*) ethanol concentration. It could be concluded that 60% (*v*/*v*) ethanol aqueous solution has the most similar polarity to the antioxidants in the sample, based on the principle of “like dissolves like” [12]. The low-concentration ethanol aqueous solution has different dielectric properties on microwave energy, which affects the heat distribution in the sample, thereby may leading to a decrease in antioxidant activity and TPC of the extract [29]. On the other hand, high-concentration ethanol denatures proteins and then reduces the dissolution and the recovery of polyphenols [30]. Based on the results of the three tests, 60% (*v*/*v*) was selected as the optimal concentration of ethanol for subsequent experiments.

#### 3.1.2. Effects of Solvent-to-Sample Ratio

The solvent-to-sample ratio also influences antioxidant activity and TPC of the leaf extract of *L. polystachyus*. Under the extraction condition of 500 W, 50 °C, 60% (*v/v*) and 30 min, the TEAC value, FRAP value and TPC of the extract were measured at the solvent-to-sample ratio of 10:1, 20:1, 30:1, 40:1, 50:1 and 60:1 mL/g, respectively.

The results showed that the TEAC, FRAP and TPC values were the highest when the solvent-to-sample ratio was 30:1 mL/g (Figure 1b). With the volume of the solvent increased (10:1–30:1 mL/g), the antioxidant capacity and TPC of the extract were both elevated. When the ratio higher than 30:1, the antioxidant capacity and TPC fluctuated within a small range and basically remained stable. One possibility is that as the volume of the solvent increases, the contact surface area between the sample and the solvent expands, allowing extraction to proceed adequately [31]. Thus, 30:1 mL/g was selected as the optimum solvent-to-sample ratio.

#### 3.1.3. Effects of Extraction Temperature

Other conditions were fixed at 500 W, 60% (*v*/*v*), 30:1 mg/L and 30 min and the corresponding TEAC, FRAP and TPC values were explored when the extraction temperature was 30–70 °C and the variation trends are exhibited in Figure 1c.

There was no significant difference in TEAC values of the extracts at different extraction temperatures, while for FRAP and TPC values, they reached their maximum values at an extraction temperature of 50 °C. FRAP value gradually elevated from 30 °C to 50 °C and slightly decreased when it was higher than 50 °C. The variation trend of TPC was consistent with that of FRAP value. The rising temperature usually increases the diffusion rate of the solvent into the sample and the desorption of the target compounds into the solvent [31]. However, when the temperature is higher than the appropriate value, the extracted antioxidants might break down or degrade [32]. As a result, 50 °C was the optimal extraction temperature.

#### 3.1.4. Effects of Microwave Power

Microwave power is a crucial factor affecting the efficiency of microwave-assisted extraction. At the parameters of 60% (*v*/*v*), 30:1 mg/L, 50 °C and 30 min, the influence of microwave power of 400–900 W on antioxidant capacity and TPC of the extract were considered (Figure 1d).

The FRAP value increased continuously from microwave power of 400 W and reached the highest value at 700 W, among which the FRAP value at 600 W was close to that at 700 W. When the microwave power was higher than 700 W, the FRAP value decreased. For TEAC and TPC values, the trend was parabolic and the peak was 600 W microwave power. The increase of the microwave power could strengthen the molecular interaction between the electromagnetic field and sample and improve the extraction efficiency [31]. However, the continued increase in microwave power might cause the degradation of the target antioxidants [12]. Therefore, microwave power of 600 W was used for response surface experiments.

#### 3.1.5. Effects of Extraction Time

With the optimized values of previous experiments as extraction conditions (600 W, 50 °C, 30:1 mg/L and 60% *v*/*v*), the extraction efficiency was investigated when the extraction time was 10–60 min.

As shown in Figure 1e, the TEAC, FRAP and TPC values at the extraction time of 20 min were higher than those at the extraction time of 10 min. As the extraction time exceeded 20 min, the antioxidant activity and TPC of the extracts decreased slightly and there was no significant difference between 30–60 min. When the extraction time was less than 20 min, the microwave action time was short and the extraction might be insufficient. However, the prolonged high temperature due to the excessive extraction time might cause the degradation of antioxidants [33]. On the other hand, the microwave equipment worked intermittently in order to maintain a constant temperature, so the difference in actual microwave time might be smaller and the long extraction time resulted in more energy consumption. Hence, 20 min was chosen to be the optimal extraction time.

### 3.2. Analysis of Response Surface Methodology (RSM) Experiments

#### 3.2.1. Results of Central Composite Design (CCD)

According to the results of single-factor tests, ethanol concentration, solvent-to-sample ratio and extraction time were selected as the extraction conditions optimized by RSM, because they contributed more to the changes of TEAC, FRAP and TPC values. The center point of CCD was set as solvent-to-sample ratio of 30:1 mL/g, extraction time of 20 min and ethanol concentration of 60% (*v*/*v*) and the microwave power and extraction temperature were fixed at 600 W and 50 °C. The design of 20 runs and the corresponding actual response values of FRAP, TEAC and TPC are shown in Table 3. For the antioxidant activity of *L. polystachyus* leaf extract, the TEAC values ranged from 445.12 to 622.61 μmol Trolox/g DW and the FRAP values ranged from 284.49 to 390.85 µmol Fe(II)/g DW. In addition, the TPC values of *L. polystachyus* leaf extract varied from 108.70 to 135.79 mg GAE/g DW.

#### 3.2.2. Fitting the Models and Statistical Analysis

The data in Table 3 were analyzed by multiple regression fitting and three quadratic multinomial equations describing the relationship between the three variables and TEAC, FRAP and TPC were obtained (excluding insignificant coefficients):

Y_FRAP_ = 378.95 + 7.73*X*_2_ − 22.86*X*_3_ + 11.59*X*_1_*X*_2_ + 10.85*X*_2_*X*_3_ − 7.55*X*_1_^2^ − 10.58*X*_2_^2^ − 18.43*X*_3_^2^, (2)

Y_TEAC_ = 603.65 + 33.97*X*_1_ + 7.83*X*_3_ + 15.95*X*_1_*X*_2_− 37.90*X*_1_^2^ − 8.01*X*_2_^2^ − 8.36*X*_3_^2^, (3)

Y_TPC_ = 132.65 + 3.13*X*_1_ + 2.03*X*_2_ + 2.35*X*_1_*X*_2_ − 2.13*X*_1_*X*_3_ + 5.03*X*_2_*X*_3_ − 3.78*X*_1_^2^ − 3.79*X*_2_^2^ − 4.70*X*_3_^2^, (4)

where Y refers to the response values of TEAC, FRAP and TPC of the extract, X_1_, X_2_, X_3_ are solvent-to-sample ratio, extraction time and ethanol concentration, respectively.

The analysis of variance (ANOVA) was performed on the three quadratic polynomial regression models with response values of FRAP, TEAC and TPC and the results showed that the three models were extremely significant (*p* < 0.0001), with *F* values of 27.52, 31.18 and 28.46, respectively (Table 4). The coefficient of determination (*R*^2^) of FRAP, TEAC and TPC fitting models were 0.9612, 0.9656 and 0.9624, respectively, all greater than 0.80, suggesting that 96.12%, 96.56% and 96.24% of the data could be described by these three models. The adjusted *R*^2^ (*R*^2^_Adj_) of FRAP, TEAC and TPC models were 0.9263, 0.9346 and 0.9286, respectively, that is, there were only 3.49%, 3.10% and 3.38% significant differences between the predicted values and the actual values, which proved the reliability of the model. In addition, the ‘Lack of Fit’ terms were not significant (*p* > 0.05), indicating that the models were appropriate.

#### 3.2.3. Effects of Independent Variables on Antioxidant Activity

The effects of three independent variables, that is, solvent-to-sample ratio (X_1_), extraction time (X_2_) and ethanol concentration (X_3_), on the antioxidant activity of *L. polystachyus* leaf extract detected by FRAP and TEAC assays are shown in Equations (2), (3) and Table 5.

**Figure 2:**
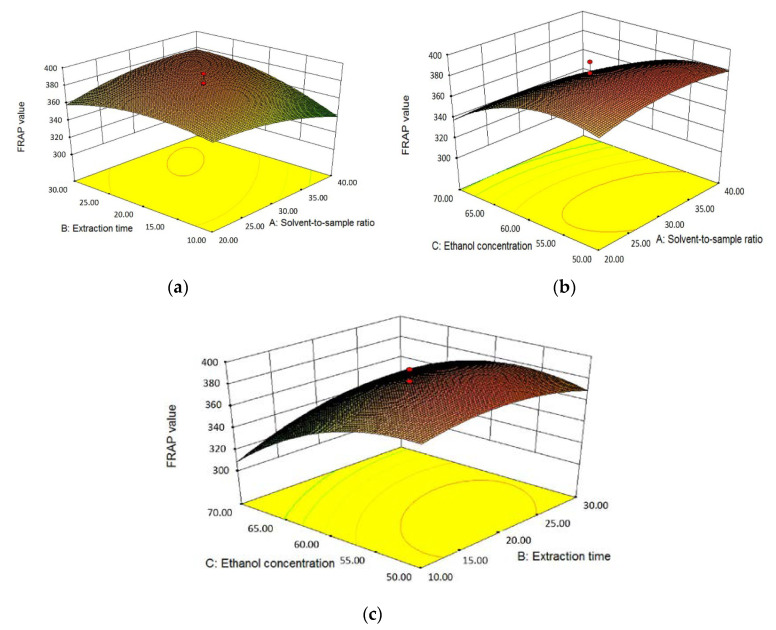
The three-dimensional response surface plots for the effects of solvent-to-sample ratio/extraction time (**a**); solvent-to-sample ratio/ethanol concentration (**b**); and ethanol concentration/extraction time (**c**) on the FRAP values.

According to the estimated coefficients of the fitting models and their statistical differences based on ANOVA, it was shown that for FRAP values, the linear term and quadratic term of ethanol concentration (X_3_ and X_3_^2^), the quadratic terms of extraction time (X_2_^2^) had negative and extremely significant influences (*p* < 0.0001). The linear term of extraction time (X_2_), as well as its interaction terms with solvent-to-sample ratio (X_1_X_2_) and ethanol concentration (X_2_X_3_) had positive and very significant effects (*p* < 0.01) on FRAP values, while the quadratic term of solvent-to-sample ratio had a very significant negative effect. In addition, the three-dimensional response surface plots visually described the relationship between FRAP values and three independent variables (Figure 2). The Figure 2a shows that the FRAP value first increased with the increase of solvent-to-sample ratio and extraction time and then decreased when it was close to the solvent-to-sample ratio of 40:1 mL/g and the time of 30 min, with the ethanol concentration fixed at 60% (*v*/*v*). The contour plot was elliptic, which indicated that there was a strong interaction between solvent-to-sample ratio and extraction time [34]. According to Figure 2b, the ethanol concentration also had a quadratic effect on FRAP, when the extraction time was 20 min. When the ethanol concentration rose from 50% to about 55% (*v*/*v*), the maximum FRAP value was obtained and then the FRAP values apparently declined until the concentration reached 70% (*v*/*v*). When the solvent-to-sample ratio remained 30:1 mL/g, the influence trends of extraction time and ethanol concentration on FRAP in Figure 2c were similar to those in Figure 2a,b.

**Figure 3:**
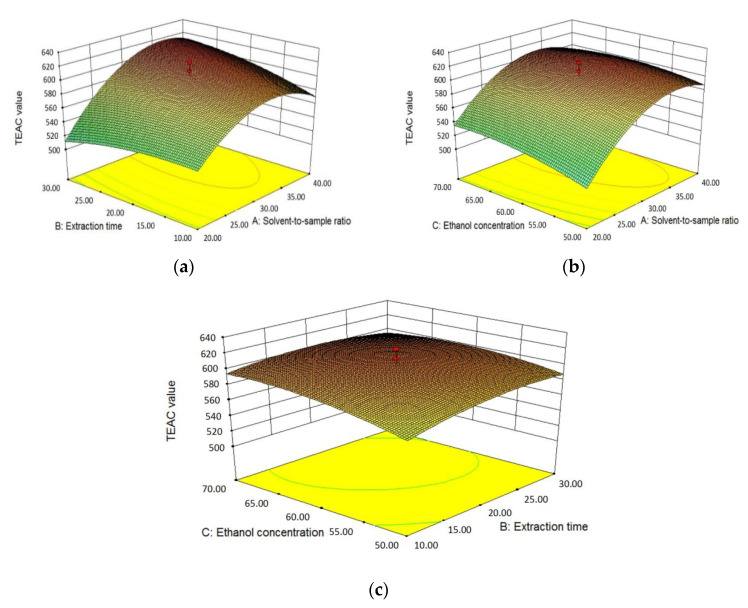
The three-dimensional response surface plots for the effects of solvent-to-sample ratio/extraction time (**a**); solvent-to-sample ratio/ethanol concentration (**b**); and ethanol concentration/extraction time (**c**) on the TEAC values.

In term of TEAC, as shown in Table 4, the solvent-to-sample ratio had both extremely significant linear and quadratic effects (*p* < 0.0001) on TEAC values and the linear term (X_1_) had a positive effect, while the quadratic term (X_1_^2^) had a negative effect. Similarly, the ethanol concentration had a significant positive linear effect (X_3_, *p* < 0.05) and a significant negative quadratic effect (X_3_^2^, *p* < 0.05) on TEAC. The linear term of extraction time (X_2_) and the cross product of extraction time and solvent-to-sample ratio (X_1_X_2_) significantly influenced TEAC, which were positive and negative, respectively. Figure 3 showed the three-dimensional response surface plots of TEAC values and three independent variables. As shown in Figure 3a, when the ethanol concentration was 60% (*v*/*v*), the TEAC value increased significantly with the increase of solvent-to-sample ratio and then decreased slightly. The effect of extraction time on TEAC was relatively gentle. When the solvent-to-sample ratio was relatively high, TEAC showed an upward trend with the extension of time from 10 to 30 min and reached the maximum when approaching 30 min. However, when the solvent-to-sample ratio was at a low value, TEAC showed a downward trend over time. Thus, the solvent-to-sample ratio and extraction time had obvious interaction, which could also be seen from the elliptic contour plot and the results were consistent with ANOVA. Figure 3b displayed that when the extraction time was fixed at 20 min, TEAC value increased slightly as the ethanol concentration increased from 50% to around 65% (*v*/*v*) and then decreased. Likewise, the effects of extraction time and ethanol concentration on TEAC in Figure 3c were the same as those in Figure 3a,b, with the solvent-to-sample ratio maintaining at 30:1 mL/g.

To sum up, the independent variables of solvent-to-sample ratio, extraction time and ethanol concentration not only had direct effects on the antioxidant activity of *L. polystachyus* leaf extract but also had more significant quadratic effects. Additionally, the influences of solvent-to-sample ratio and ethanol concentration on antioxidant activity were stronger than that of extraction time.

#### 3.2.4. Effects of Independent Variables on TPC

The effects of solvent-to-sample ratio (X_1_), extraction time (X_2_) and ethanol concentration (X_3_) on TPC values of *L. polystachyus* leaf extracts obtained from 20 experiments were fitted by a quadratic regression equation, as shown in Equation (4). Meanwhile, the estimated coefficients and the results of ANOVA are shown in Table 4. The solvent-to-sample ratio (X_1_) and the extraction time (X_2_) positively and directly affected the TPC values at *p* < 0.01. The cross products of the three independent variables all had significant effects on TPC values, among which the interaction terms of extraction time and other two variables (X_1_X_2_ and X_2_X_3_) had very significant positive influences on TPC values (*p* < 0.01). All the quadratic terms (X_1_^2^, X_2_^2^ and X_3_^2^) had extremely significant (*p* < 0.0001) negative effects on TPC values.

**Figure 4:**
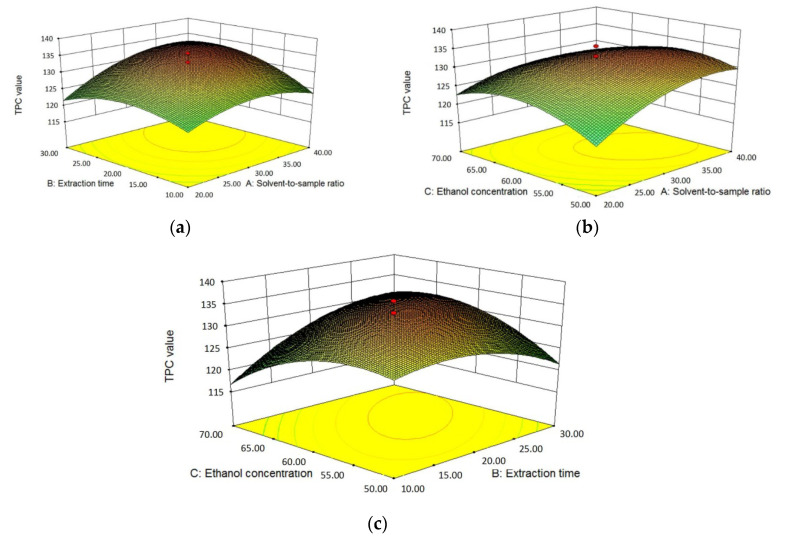
The three-dimensional response surface plots for the effects of solvent-to-sample ratio/extraction time (**a**); solvent-to-sample ratio/ethanol concentration (**b**); and ethanol concentration/extraction time (**c**) on the TPC values.

Furthermore, the interactions among solvent-to-sample ratio, extraction time, ethanol concentration and their effects on the TPC value were plotted in Figure 4. In Figure 4a, when the ethanol concentration was fixed at 60% (*v*/*v*), both the solvent-to-sample ratio and the ethanol concentration had quadratic effects on TPC values and the peak value of TPC was at about 35 mg/L and 25 min. Figure 4b showed the interaction of solvent-to-sample ratio and ethanol concentration on the TPC at an extraction time of 20 min. The TPC elevated with the increase of solvent-to-sample ratio but as the further increase of ratio from 35:1 to 40:1 mL/g, the elevation was limited. The effect of ethanol concentration on TPC was similar to that of solvent-to-sample ratio and the maximum value of TPC was obtained at about 58% (*v*/*v*). In Figure 4c, the trend of the influences of extraction time and ethanol concentration on TPC were close to those in Figure 4a,b. Hence, these independent variables not only had the direct linear effects on the TPC values but also presented a significant quadratic effect. There were interactions between these three variables, among which the interaction term of extraction time and ethanol concentration had the most significant effect on TPC values. What’s more, the effect of solvent-to-sample ratio on TPC was stronger than those of extraction time and ethanol concentration.

#### 3.2.5. Verification of the Model

The optimal microwave-assisted extraction conditions for the highest antioxidant activities and total phenol content of *L. polystachyus* leaf extract were obtained by RSM experiment and the predicted conditions were validated (Table 6). Under the optimal extraction conditions—microwave power of 600 W, extraction temperature of 50 °C, the ethanol concentration of 58.43% (*v*/*v*), solvent-to-sample ratio of 35.39:1 mL/g and extraction time of 25.26 min, the extracts had a FRAP value of 381.29 ± 4.42 μM Fe(II)/g DW, a TEAC value of 613.11 ± 9.32 μM Trolox/g DW and a TPC value of 135.94 ± 0.52 mg GAE/g DW. The actual results were close to the predicted values of 383.23 μM Fe(II)/g DW, 614.52 μM Trolox/g DW and 133.62 mg GAE/g DW, which confirmed the reliability and accuracy of the fitted model. Further, the traditional maceration method was also conducted to extract antioxidants from *L. polystachyus* leaf (Table 6). The FRAP, TEAC and TPC values of the extracts were 281.82 ± 9.21 μM Fe(II)/g DW, 540.8 ± 7.51 μM Trolox/g DW and 90.59 ± 0.67 mg GAE/g DW. In contrast, microwave-assisted extraction elevated the extraction rate by 35.30%, 13.35% and 50.05%, respectively and the extraction efficiency was also improved.

### 3.3. Identification of Phenolic Compounds in L. Polystachyus Leaf Extract

**Figure 5:**
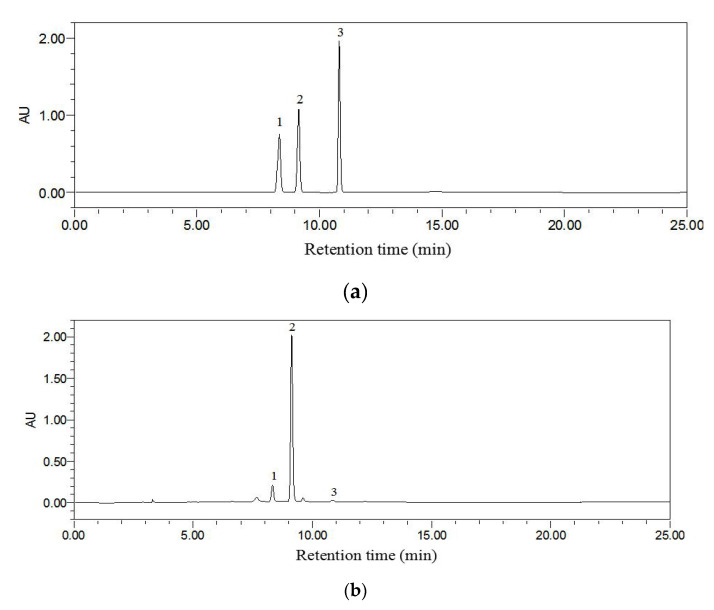
The high-performance liquid chromatography with diode array detection (HPLC-DAD) chromatograms at 283 nm of standards (**a**); and *L. polystachyus* leaf extract obtained under optimal extraction condition (**b**). The labeled number represents the compound: 1, phlorizin; 2, trilobatin; and 3, phloretin.

Phenolic compounds make a great contribution to antioxidant activity, so the identification of phenolics could help to clarify the biological activities of *L*. *polystachyus* leaf extract. The main phenolic compounds of *L*. *polystachyus* leaf extract obtained under optimal extraction condition were identified and quantified by HPLC-DAD, including phlorizin, trilobatin and phloretin. The chromatograms at 283 nm of the standard compounds and *L*. *polystachyus* leaf extract are shown in Figure 5a,b. Among them, the content of trilobatin was the highest in the extract (164.38 ± 0.15 mg/g DW), followed by phlorizin (23.87 ± 0.19 mg/g DW) and phloretin (1.44 ± 0.01 mg/g DW) (Table 7). The antioxidant activity of *L*. *polystachyus* leaf extract might be attributed to the combined action of these compounds. Additionally, phlorizin, trilobatin and phloretin have anti-inflammatory, antimicrobial, anti-diabetic, anticancer, cardioprotective and hepatoprotective activities, suggesting that *L*. *polystachyus* leaf extract might also have these potential health benefits [5,6,35,36,37,38,39].

## 4. Conclusions

The optimum microwave-assisted extraction condition of antioxidants from *L. polystachyus* leaf was studied. Three independent variables (ethanol concentration, solvent-to-sample ratio and extraction time) with great influence on the extraction efficiency were acquired through single factor tests and these parameters were further optimized by RSM based on CCD design. The quadratic models obtained by RSM were accurate and reliable, with high *R*^2^ and *R*^2^_Adj_. The extraction conditions for the highest in vitro antioxidant activity and TPC were as follows—microwave power of 600 W, extraction temperature of 50 °C, solvent-to-sample ratio of 35.39:1 mL/g, extraction time of 25.26 min and ethanol concentration of 58.43% (*v*/*v*). Under this optimal condition, the FRAP value of the extract was 381.29 ± 4.42 μM Fe(II)/g DW, the TEAC value was 613.11 ± 9.32 μM Trolox/g DW and the TPC value was 135.94 ± 0.52 mg GAE/g DW. In addition, compared with the traditional maceration method, the microwave-assisted method effectively improved the efficiency of extracting antioxidants from *L. polystachyus* leaf. Further, the main phenolic compounds of *L. polystachyus* leaf, including phlorizin, phloretin and trilobatin, were analyzed qualitatively and quantitatively by HPLC-DAD and they might contribute to the antioxidant activity of the extract. In the future, the crude extract could be developed to into antioxidant food additives for industrial production and the extract and its bioactive compounds might be the promising participants in antioxidant functional food for the prevention and management of oxidative stress-related diseases.

## Figures and Tables

**Table 1:** Independent variables and code levels of central composite design (CCD).

Independent Variable	Units	Code levels				
		−1.68	−1	0	1	1.68
Solvent-to-sample ratio (*X*_1_)	mL/g	13.18	20	30	40	46.82
Extraction time (*X*_2_)	min	3.18	10	20	30	36.82
Ethanol concentration (*X*_3_)	%, *v*/*v*	43.18	50	60	70	76.82

**Table 2:** The composition of the mobile phase and the conditions of gradient elution.

Time (min)	Solution A	Solution B
0	35	65
8	70	30
15	60	40
25	35	65

**Table 3:** The design and the corresponding actual values of CCD.

Run	X_1_	X_2_	X_3_	Y (Actual Response Values)		
	Solvent-to-Sample Ratio	Extraction Time	Ethanol Concentration	FRAP	TEAC	TPC
	mL/g	min	%, *v*/*v*	µmoL Fe(II)/g DW	μmoL Trolox/g DW	mg GAE/g DW
1	30 (0)	3.18 (−1.68)	60 (0)	329.33	567.71	117.85
2	40 (1)	10 (−1)	70 (1)	284.49	582.04	112.11
3	20 (−1)	10 (−1)	70 (1)	325.84	539.27	112.94
4	20 (−1)	30 (1)	50 (−1)	340.12	479.63	108.70
5	30 (0)	20 (0)	43.18 (−1.68)	372.13	574.74	119.58
6	30 (0)	20 (0)	76.82 (1.68)	285.69	591.00	120.48
7	30 (0)	20 (0)	60 (0)	378.74	593.92	131.03
8	20 (−1)	30 (1)	70 (1)	333.03	514.36	123.67
9	30 (0)	20 (0)	60 (0)	380.39	622.61	131.77
10	40 (1)	10 (−1)	50 (−1)	359.64	549.56	125.76
11	30 (0)	20 (0)	60 (0)	379.84	596.59	132.22
12	40 (1)	30 (1)	70 (1)	336.88	593.50	127.40
13	30 (0)	20 (0)	60 (0)	373.23	598.07	133.08
14	30 (0)	36.82 (1.68)	60 (0)	372.89	600.03	127.33
15	46.82 (1.68)	20 (0)	60 (0)	359.37	553.54	128.57
16	30 (0)	20 (0)	60 (0)	369.93	610.85	131.77
17	20 (−1)	10 (−1)	50 (−1)	377.50	515.30	122.92
18	40 (1)	30 (1)	50 (−1)	369.78	605.10	125.76
19	30 (0)	20 (0)	60 (0)	390.85	598.88	135.79
20	13.18 (−1.68)	20 (0)	60 (0)	360.03	445.12	116.71

**Table 4:** The analysis of variance (ANOVA) of the quadratic models.

Response Value	Source	Sum of Squares	df	Mean Square	F Value	*p*-Value Prob > *F*	Significance
FRAP	Model	16,692.36	9	1854.71	27.52	<0.0001	significant
	Residual	673.94	10	67.39			
	Lack of Fit	415.41	5	83.08	1.61	0.3077	not significant
	Pure Error	258.53	5	51.71			
	Cor Total	17,366.30	19				
	*R* ^2^	0.9612					
	*R* ^2^_Adj_	0.9263					
	C.V.%	2.32					
TEAC	Model	40,446.63	9	4494.07	31.18	<0.0001	
	Residual	1441.35	10	144.13			
	Lack of Fit	831.80	5	166.36	1.36	0.3707	not significant
	Pure Error	609.55	5	121.91			
	Cor Total	41,887.98	19				
	*R* ^2^	0.9656					
	*R* ^2^_Adj_	0.9346					
	C.V.%	2.12					
TPC	Model	1086.89	9	120.77	28.46	<0.0001	significant
	Residual	42.43	10	4.24			
	Lack of Fit	28.02	5	5.60	1.94	0.2416	not significant
	Pure Error	14.41	5	2.88			
	Cor Total	1129.32	19				
	*R* ^2^	0.9624					
	*R* ^2^_Adj_	0.9286					
	C.V.%	1.66					

**Table 5:** The estimated coefficients of quadratic models and their statistical significance.

Model Parameter	FRAP		TEAC		TPC	
	Coefficient	*p*-Value	Coefficient	*p*-Value	Coefficient	*p*-Value
X_1_	−1.96	0.3978 ^d^	33.97	<0.0001 ^a^	3.13	0.0002 ^a^
X_2_	7.73	0.0059 ^b^	4.45	0.2007 ^d^	2.03	0.0045 ^b^
X_3_	−22.86	<0.0001 ^a^	7.83	0.0367 ^c^	−0.40	0.4862 ^d^
X_1_X_2_	11.59	0.0025 ^b^	15.95	0.0037 ^b^	2.35	0.0091 ^b^
X_1_X_3_	−6.16	0.0596 ^d^	−4.73	0.2914 ^d^	−2.13	0.0154 ^c^
X_2_X_3_	10.85	0.0039 ^b^	−4.17	0.3495 ^d^	5.03	<0.0001 ^a^
X_1_^2^	−7.55	0.0058 ^b^	−37.90	<0.0001 ^a^	−3.78	<0.0001 ^a^
X_2_^2^	−10.58	0.0006 ^a^	−8.01	0.0298 ^c^	−3.79	<0.0001 ^a^
X_3_^2^	−18.43	<0.0001 ^a^	−8.36	0.0246 ^c^	−4.70	<0.0001 ^a^
Intercept	378.95		603.65		132.65	

^a^ Extremely significant (*p* < 0.001); ^b^ Very significant (*p* < 0.01); ^c^ Significant (*p* < 0.05); ^d^ Not significant (*p* > 0.05).

**Table 6:** Comparison of the response values of actual experiment, fitted model prediction and maceration method.

Response Values	Extraction Methods		
	Predicted	Actual	Maceration
FRAP (μM Fe(II)/g DW)	383.23	381.29 ± 4.42	281.82 ± 9.21
TEAC (μM Trolox/g DW)	614.52	613.11 ± 9.32	540.8 ± 7.51
TPC (mg GAE/g DW)	133.62	135.94 ± 0.52	90.59 ± 0.67

**Table 7:** The contents of phenolic compounds in *L. polystachyus* leaf extract obtained under the optimal conditions.

Number	Phenolic Compounds	Retention Time (min)	Maximum Absorption (nm)	Contents (mg/g DW)
1	Phlorizin	8.33	284.5	23.87 ± 0.19
2	Trilobatin	9.13	282.1	164.38 ± 0.15
3	Phloretin	10.78	286.9	1.44 ± 0.01
